# Oxidative Stress in an African Ground Squirrel, a Case of Healthy Aging and Reproduction

**DOI:** 10.3390/antiox13111401

**Published:** 2024-11-15

**Authors:** Paul Juan Jacobs, Sjoerd Vos, Chelsea E. Bishop, Daniel William Hart, Nigel Charles Bennett, Jane M. Waterman

**Affiliations:** 1 Department of Zoology and Entomology, Mammal Research Institute, University of Pretoria, Pretoria 0002, South Africa; daniel.hart@zoology.up.ac.za (D.W.H.); ncbennett@zoology.up.ac.za (N.C.B.); jane.waterman@umanitoba.ca (J.M.W.); 2 Department of Biological Sciences, University of Manitoba, Winnipeg, MB R3T 2N2, Canada; voss1@myumanitoba.ca (S.V.); bishopc2@myumanitoba.ca (C.E.B.)

**Keywords:** life-history strategies, *Xerus inauris*, oxidative stress, aging, reproductive health

## Abstract

Oxidative stress plays a crucial role in mediating life-history processes, where it can compromise survival and reproduction through harmful alterations to DNA, lipids, and proteins. In this study, we investigated oxidative stress in Cape ground squirrels (*Xerus inauris*), a longer-lived African ground squirrel species with a high reproductive skew and unique life history strategies. We measured oxidative stress as total antioxidant capacity (TAC), total oxidant status (TOS), and an oxidative stress index (OSI) in blood plasma from individuals of approximately known ages. Our results reveal a distinct pattern of decreasing oxidative stress with age, consistent across both sexes. Females exhibited lower OSI and TOS levels than males. Males employing different life-history strategies, namely natal (staying at home), had significantly lower oxidative stress compared to the band (roaming male groups), likely due to variations in metabolic rate, activity, and feeding rates. However, both strategies exhibited reduced oxidative stress with age, though the underlying mechanisms require further investigation. We propose that selection pressures favoring survival contributed to the observed reduction in oxidative stress with age, potentially maximizing lifetime reproductive success in this species.

## 1. Introduction

Life history theory investigates how animals allocate limited resources between aging, survival, and reproduction, shaping their overall evolutionary fitness [1,2,3]. Traditionally, life history and aging biology suggest that trade-offs between reproduction and longevity drive the evolution of aging rates, lifespans, and reproductive success [4,5,6]. This suggestion is based on the concept that reproductive success post-maturity often comes at the cost of accelerated aging, and vice versa. However, animals have evolved diverse behavioral and physiological strategies to navigate these trade-offs [7]. For example, some species invest heavily in maintaining their own body condition to enhance survival (somatic maintenance or self-maintenance strategy), while others prioritize reproductive success (reproductive effort or reproductive investment strategy) [3,8]. Moreover, reproductive strategies (for example dominant and subordinate strategies) and cooperative behaviors such as group living can help mitigate these life-history trade-offs [9,10]. Dominant reproductive tactics occur when males or females gain priority access to mates, often through social dominance or physical competition [11]. In contrast, subordinate strategies involve avoiding direct competition with dominant individuals [11]. Subordinate animals may attempt to mate opportunistically, such as when dominant breeders are distracted or absent [12]. Both tactics aim to maximize reproductive success, and the choice of strategy can depend on factors like social status, environmental conditions, developmental stage, or population density [12,13,14,15]. In such species, increased body condition through social support may enhance reproductive success, even in aging individuals [16]. Certain physiological adaptations, including those that bolster immunity during reproduction, may further improve survival, longevity, and overall fitness [17]. Nonetheless, the underlying mitigating mechanisms of these trade-offs remain poorly understood [3,18,19,20].

Oxidative stress is thought to play a central role in balancing the physiological trade-offs between survival, reproduction, and aging [21,22,23,24,25,26]. This condition arises when the production of reactive oxygen species (ROS)—highly reactive molecules produced naturally during metabolism—exceeds the body’s antioxidant defenses [27,28,29,30]. When ROS production is in excess past normal physiological levels, ROS can cause cellular damage to DNA, lipids, and proteins, which may reduce an organism’s ability to survive and reproduce [27,28,29,30]. This impact of oxidative stress is particularly significant during reproduction, as the heightened metabolic demands of reproduction can increase ROS production, imposing unavoidable costs on survival [18,21,31,32,33,34]. Given this increase, oxidative stress may represent a “proximate cost” of reproduction—a physiological consequence that organisms must balance when investing energy in reproduction [19,35,36,37,38]. Adding to the complexity, oxidative stress and aging are closely intertwined [39,40,41]. Although oxidative stress generally increases with age, some species, like the naked mole-rat (Heterocephalus glaber), have evolved mechanisms that mitigate this damage, potentially influencing longevity [40,42,43,44,45]. Despite the recognized role of oxidative stress in life-history trade-offs, there are relatively few studies examining how alternative reproductive strategies over an animal’s lifespan (age) can impact whole-body oxidative stress.

The Cape ground squirrel (*Xerus inauris*) presents an intriguing model to study the relationship between oxidative stress, aging, and reproduction. This non-hibernating [46,47] group-living species with a promiscuous and somatic maintenance mating system is hypothesized to use various behavioral and physiological strategies to offset life-history trade-offs [48,49]. Cape ground squirrels exhibit high reproductive skew [50,51,52]. Male squirrels employ two distinct reproductive tactics or alternate reproductive tactics: band males, who join same-sex roving groups in search of estrous females [53], and natal males, who, at maturity, delay dispersal and remain with their natal group and provide alloparental care [50,51]. Despite these differences, reproductive success is similar between the two alternate reproductive tactics [50], and intriguingly, reproduction in this species does not appear to compromise body condition [49,54]. In fact, reproductive success increases with age in male Cape ground squirrels [55,56]. Given the unique life history of this species, oxidative stress may reveal important physiological mechanisms that help to explain these life history trade-offs.

This study aimed to explore how oxidative stress varies with body condition—an indicator of diet and overall nutritional health—and age in wild-caught male and female Cape ground squirrels. Specifically, we examined oxidative stress in both band males and natal males by measuring total antioxidant capacity (TAC), total oxidative status (TOS), and their ratio, known as the oxidative stress index (OSI). Since age and body condition are influential factors in reproduction for this species, with older males generally achieving higher reproductive success and body condition differing between reproductive strategies, we tested specific hypotheses on how these variables impact oxidative stress. We hypothesized that females would exhibit lower oxidative stress than males, consistent with findings from previous studies [57,58]. We predicted that oxidative stress would increase with age as predicted by the metabolic theory of aging [59]. Additionally, among males, we expected band and natal males to display similar oxidative stress levels, reflecting their comparable reproductive success.

## 2. Materials and Methods

### 2.1. Ethics Statement

Experimental procedures adhered to the recommendations outlined in the National Institutes of Health Guide for the Care and Use of Laboratory Animals and the American Society of Mammalogists Animal Care guidelines [60,61].

### 2.2. Study Site

Free-ranging Cape ground squirrels were captured at the S.A. Lombard Nature Reserve (4600 ha), located 18 km northwest of Bloemhof, South Africa (27°35′ S, 25°23′ E). The study was conducted from May to July 2024. Blood samples for oxidative stress analyses, along with age and morphological measurements to assess body condition, were collected from the animals. The reserve’s habitat consists of a floodplain with dry Cymbopogon-Themeda grassland and black soil turfveld, interspersed with bush and pan areas [62]. During the 2023/2024 period (1 July to 30 June), the total rainfall was 465 mm, with 304.8 mm falling between January and April. March had below-average rainfall, recording only 10 mm compared to the usual 71 mm, suggesting a slight reduction in primary productivity.

### 2.3. Study Species, Trapping, Body Condition, and Reproductive Determination

Cape ground squirrels were trapped using Tomahawk live traps (15 × 15 × 50 cm) baited with peanut butter and bird seed [48]. Trapping was conducted 2–4 times daily (approx. 70 traps per round), between 07:00 and 17:30. Traps were shaded and checked every hour to minimize heat stress. Each squirrel was marked with a pit tag (Shenzhen XCC RFID Technology Co., Ltd., Shenzhen, China) for permanent identification and a dorsal black hair dye mark (Rodol D; Lowenstein and Sons Inc., Brooklyn, NY, USA) for identification from a distance. This population has been monitored yearly since 2002 resulting in long-term life-history data for all animals.

We captured 42 animals (26 males and 16 females) with known ages for this study. For each individual, we measured body mass to the nearest 0.5 g using a Pesola Spring scale (Pesola AG, Baar, Switzerland) and recorded spine length from the base of the skull to the base of the tail using a tape measure. We also assessed each animal’s reproductive condition. To evaluate body condition, we followed the methods outlined by Schulte-Hostedde, et al. [63]. In brief, we calculated a body condition index using the residuals from the ordinary least squares regression of spine length and body mass, where individuals with better body condition exhibit positive residual values (Table S1) [63].

Age determination for all animals in this study followed established methods, and all individuals were of reproductive age [49,50]. Among the 16 female squirrels sampled, 4 showed signs of oestrus or pregnancy. However, due to the small sample size of these reproductively active females, we did not perform comparisons based on reproductive state, though we included them in the age analyses. The ages of females ranged from two to eight years. Males were classified based on their social behavior into two groups: natal males (those remaining within their family groups and delay dispersal) and band males (those that had dispersed to form or join all-male groups). Natal males typically associate and sleep with family members, whereas band males sleep with other dispersed adult males [48,64]. Natal males will eventually disperse into these bands, where the maximum age of delayed dispersal in natal males is 5 years [50,53,64]. Because band males typically dispersed onto our study site from other areas, their exact ages were not known. Instead, we used their tenure on the site as adults as a proxy for age. Given that natal males generally disperse around 3.5 years of age (range 1–5 years; O’Brien, Waterman, and Bennett [64]), this proxy may underestimate the age of some band males. In this study, estimated ages for band males ranged from one to seven years, while natal males ranged from one to four years.

### 2.4. Blood Sample Collection and Storage

To collect blood, we transferred individuals from the live traps into cotton handling bags designed to reduce movement and minimize stress while handling, allowing us to collect blood quickly (<5 min) without anesthesia [65]. Animals were then released at their site of capture. Approximately 1 mL of whole blood was collected from the femoral vein using a sterile 26-gauge needle and syringe within the first two minutes of handling. The blood was transferred into microcentrifuge tubes containing heparin to prevent clotting. Samples were centrifuged at 6000 rpm for 10 min at the field lab and stored at −20 °C. The samples were later transported to the University of Pretoria, where they were kept at −70 °C to −80 °C until oxidative stress analysis.

### 2.5. Reagents

All chemicals and reagents used in the study, unless otherwise stated, were obtained from Merck (Pty) Ltd., Johannesburg, Gauteng, South Africa. Ammonium iron (II) sulfate hexahydrate (215406; CAS 7783-85-9), Xylenol orange disodium salt (52097; CAS 1611-35-4), Hydrogen Peroxide 100 vol (1053872; CAS 7722-84-1), Sulfuric Acid (258105; CAS 7664-93-9), o-Dianisidine dihydrochloride (D9154; CAS 20325-40-0), Glycerol (G5516; CAS 56-81-5), and Sodium Chloride (746398; CAS 7647-14-5).

### 2.6. TAC Assay

Plasma TAC was measured using a commercial total antioxidant capacity it (Item 709100, Cayman Chemical Co., Ann Arbor, MI, USA). This assay quantifies the inhibition of ABTS (2,29-Azino-di-[3-ethybenzthiazoline sulphonate] oxidation by non-enzymatic antioxidants in the sample. Oxidized ABTS is detected spectrophotometrically at 750 nm, and antioxidant capacity is expressed as micromole Trolox equivalents per liter (μmol Trolox equivalents/L). Each sample was tested in duplicate across two assay plates (repeatability: r = 0.99), with intra-assay variability at 1.78%.

### 2.7. TOS Assay

Plasma TOS levels were determined using Erel’s method, which relies on the oxidation of ferrous ions to ferric ions in the presence of oxidative species [66]. The oxidation reaction is enhanced by glycerol molecules, which are abundantly present in the reaction medium. The ferric ions form a colored complex with xylenol orange in an acidic medium, which is measured spectrophotometrically. The results are expressed as micromole hydrogen peroxide equivalent per liter (μmol hydrogen peroxide (H_2_O_2_) equivalent/L). Samples were tested in duplicate (repeatability: r = 0.99), with intra-assay variability at 2.75%.

### 2.8. OSI

The OSI was calculated as the ratio of TOS to TAC, representing an arbitrary unit of oxidative stress, as follows: OSI = [(TOS, μmol H_2_O_2_ equivalent/L)/(TAC, μmol Trolox equivalent/L)].

### 2.9. Statistical Analysis

All statistical analyses were performed using R version 4.3.3 [67]. The response variables were TOS, TAC, and OSI. For all models, the predictors included age, sex state (with separate models comparing males vs. females and band vs. natal), body condition, and their interactions. Data normality was evaluated using the Shapiro-Wilk test and homogeneity of variance was assessed with Levene’s test. Generalized linear models were fitted using the ‘glm’ function, with stepwise model selection guided by the Akaike Information Criterion. Data visualization was performed using the ggplot2 package 3.5.1 [68]. Separate generalized linear models were used to visualize the linear relationships between oxidative stress, age, and body condition across groups. The results are expressed as mean ± standard error (s.e.m), and statistical significance was set at *p* ≤ 0.05.

## 3. Results

### 3.1. Sex Differences in Oxidative Stress

**Figure 1:**
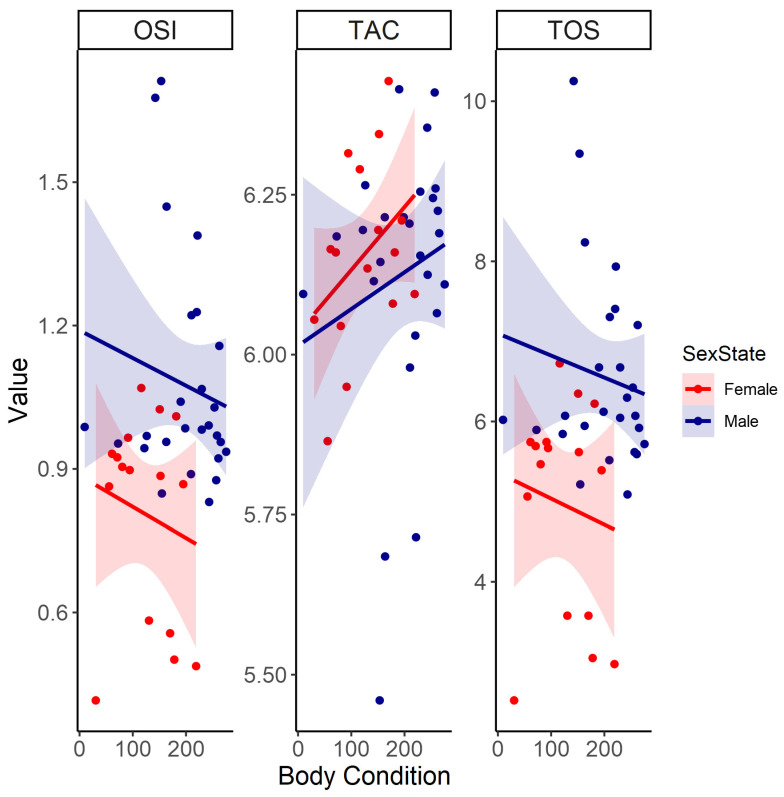
The linear relationships between body condition (body condition—residuals of size/mass regression) and oxidative variables, namely oxidative stress index (OSI), total antioxidant capacity (TAC), and total oxidant status (TOS) between female and male Cape ground squirrels (*Xerus inauris*). Red values for females and dark blue values for males. The line represents a generalized linear model regression with a Gaussian family and identity link as a representation for the statistical outputs. The shaded areas around the smoothed line represent the 95% confidence intervals, indicating the range in which the true smoothed values are likely to fall with 95% confidence.

**Figure 2:**
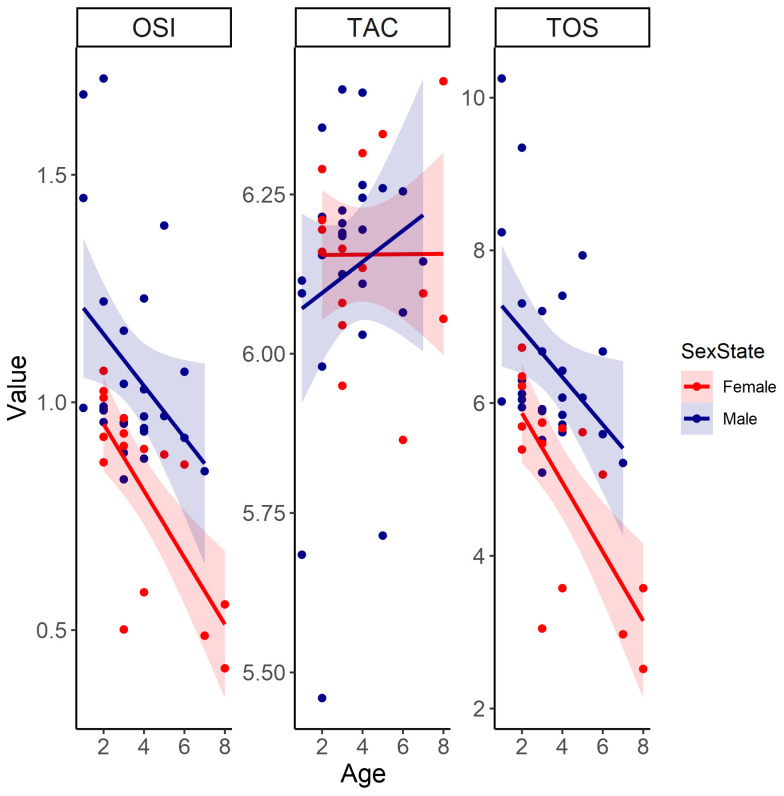
The linear relationships between age (years) and oxidative variables, namely oxidative stress index (OSI), total antioxidant capacity (TAC), and total oxidant status (TOS) between female and male Cape ground squirrels (*Xerus inauris*). Red values for females and dark blue values for males. The line represents a generalized linear model regression with a Gaussian family and identity link as a representation for the statistical outputs. The shaded areas around the smoothed line represent the 95% confidence intervals, indicating the range in which the true smoothed values are likely to fall with 95% confidence.

Oxidative stress markers, including TOS and OSI, were inversely related to body condition but were not significantly affected by body condition itself (Table 1, Figure 1). In both sexes, TOS and OSI levels decreased significantly with age and were consistently lower in females than in males (Table 1, Figure 2). Additionally, females had significantly lower OSI than males (Table 1, Figure 2). Contrastingly, TAC was not significantly influenced by any of the predictors (Table 1, Figure 1 and Figure 2).

### 3.2. Natal and Band Males

**Figure 3:**
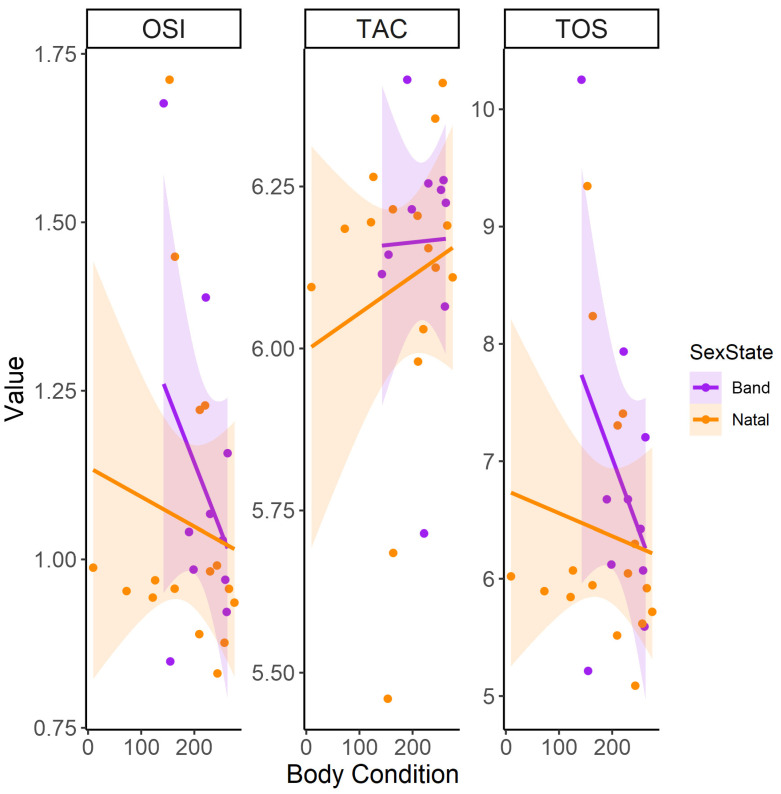
The linear relationship between body condition (body condition—residuals of size/mass regression) and oxidative variables, namely oxidative stress index (OSI), total antioxidant capacity (TAC), and total oxidant status (TOS), between band and natal Cape ground squirrel (*Xerus inauris*) males. Purple values for band males and orange values for natal males. Each line represents a generalized linear model regression with a Gaussian family and identity link as a representation of the statistical outputs. The shaded areas around the smoothed line represent the 95% confidence intervals, indicating the range in which the true smoothed values are likely to fall with 95% confidence.

**Figure 4:**
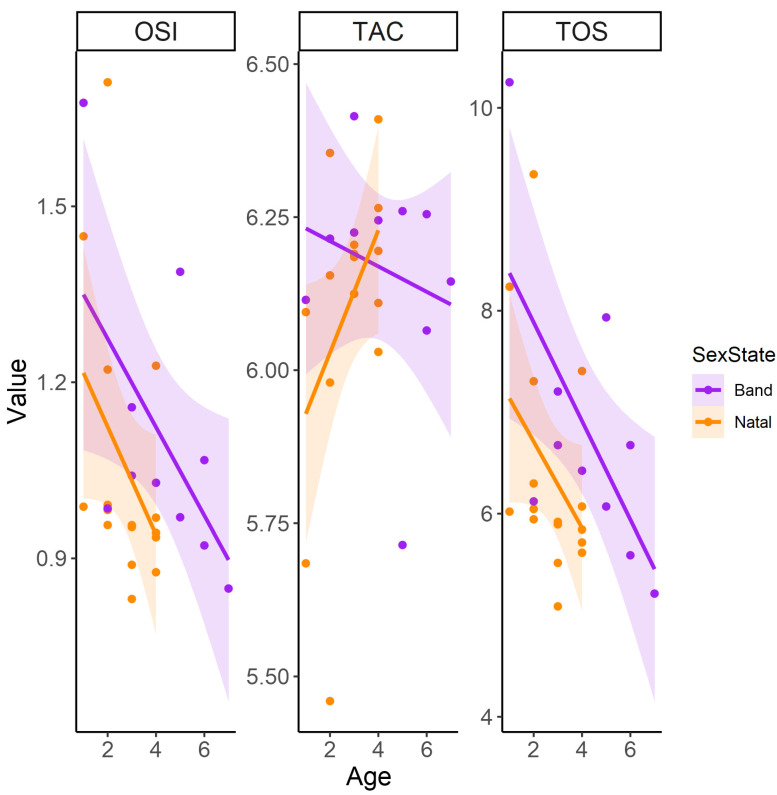
The linear relationship between age (years) and oxidative variables, namely oxidative stress index (OSI), total antioxidant capacity (TAC), and total oxidant status (TOS), between band and natal Cape ground squirrel (*Xerus inauris*) males. Purple values for the band and orange values for natal males. Each line represents a generalized linear model regression with a Gaussian family and identity link as a representation of the statistical outputs. The shaded areas around the smoothed line represent the 95% confidence intervals, indicating the range in which the true smoothed values are likely to fall with 95% confidence.

Body condition did not significantly affect oxidative markers in natal and band males, though trends indicated decreasing TOS and OSI and increasing TAC with a higher body condition in all males (Figure 3). OSI and TOS decreased significantly with age in all males, but the difference between natal and band males was not significant, although band males consistently had increased OSI levels compared to natal means across age (Table 2, Figure 4). Natal males had lower TOS levels than band males, and this effect persisted across ages but was not significant (Table 2, Figure 4). Although band males exhibited higher average TAC levels than natal males, this difference was not statistically significant (Table 2, Figure 4).

## 4. Discussion

The oxidative stress theory of aging suggests that oxidative damage accumulates over time, contributing to the aging process [69,70]. Contrary to this expectation, our data showed that circulating oxidative stress decreased with age in both sexes of ground squirrels. While plasma oxidative markers can change rapidly due to the circulation of metabolites and oxygen [71]; oxidative stress in tissues, which typically shifts more slowly [72], was not measured in this study. Our finding raises important questions about the long-term effects of oxidative stress. The observed age-related decreases in circulating oxidative stress may be due to increased enzymatic antioxidant activity [73,74] or improved cellular repair mechanisms [75,76]. The concept of hormesis—where mild stressors trigger beneficial adaptive responses—might also explain the reduction in oxidative stress with age [23,77,78]. Limited research exists on how oxidative stress changes with age in natural environments [18,79]. Studies on species like the hibernating Columbian ground squirrel (*Urocitellus columbianus*) show no significant association between age and oxidative markers [24], possibly due to recovery from hibernation-induced oxidative stress through evolutionary adaptations [80,81]. Cape ground squirrels, who do not hibernate, may experience continual self-maintenance, potentially contributing to their age-related decrease in oxidative stress.

Sex differences in oxidative stress are well-documented in mammals, with females typically exhibiting lower oxidative stress levels than males [57,58,82,83]. This difference is often attributed to estrogen, which plays a key role in antioxidant defense and can function as a potent antioxidant itself [58,84,85,86]. Additionally, females tend to produce fewer ROS due to lower NADPH-oxidase activity [86]. Our findings show that female Cape ground squirrels exhibit lower oxidative stress compared to males. Similar patterns have been observed in other mammals, such as Wistar and Spraque Dawley rats (*Rattus norvegicus*), where females show lower oxidative stress than males, a difference that diminishes in ovariectomized females [87,88]. In some group-living species, such as the Damaraland mole-rat (*Fukomys damarensis*), sex differences in oxidative stress are only observed in non-breeding individuals, but not in breeding individuals [89]. In the Natal mole-rat (*Cryptomys hottentotus natalensis*), this effect is seasonal: females exhibit lower oxidative stress than males during the summer, but not in the winter [90]. No significant sex differences have been found in the highveld mole-rat (*C. h. pretoriae*) [91]. Contrastingly, in the naked mole-rat, breeding females show a much higher OSI compared to males and non-breeding females, which all have similar OSI levels [89]. It has been postulated that naked mole rats accumulate cellular damage at an exceptionally low rate, and any increase in oxidative stress associated with reproduction is likely inconsequential due to efficient repair mechanisms compensating for an elevated oxidative stress state [43,89,92,93,94].

Male reproductive strategies also influenced oxidative stress, with distinct age-related differences observed between natal and band males. Natal males, who remain within their family groups, showed lower oxidative stress compared to band males, who dispersed into all-male groups. This difference was primarily due to variations in TOS, as TAC levels did not differ significantly between the groups. Band males face higher metabolic demands and spend less time successfully feeding compared to natal males [14]. Less time spent feeding can reduce the intake of antioxidants and minerals such as zinc and selenium, which are important for enzymatic antioxidant function [95]. Elevated metabolic demands may contribute to the observed oxidative stress differences, as increased metabolism can lead to greater free radical production [96,97]. One additional factor that may explain the lower oxidative stress observed in older band males is hormesis—the concept that exposure to mild stressors can build adaptive responses [98,99,100]. As band males experience heightened metabolic demands, they may develop physiological adaptations over time that reduce oxidative stress, potentially enhancing their reproductive success in the long term [98,99,100]. Overall, oxidative stress differences between these reproductive strategies are clear, though further research is needed to disentangle the specific contributions of metabolic rate and feeding efficiency to these observed patterns.

## 5. Conclusions

In conclusion, this study provides valuable insights into how the Cape ground squirrel employs various life-history and reproductive strategies to manage oxidative stress—an important factor that can impact longevity and reproductive success. Both male and female Cape ground squirrels appear to prioritize their survival through a variety of yet-to-be-understood physiological and/or behavioral mechanisms that may help reduce oxidative stress as they age. Additionally, we observed significant differences in oxidative stress between sexes and among male squirrels with different reproductive strategies. The variation in oxidative stress between natal males (those remaining within their birth groups) and band males (those that roam to access mates) suggests that metabolic demands and foraging behavior influence oxidative balance. Different male reproductive strategies may result in varying levels of oxidative stress, potentially affecting survival differently, even if reproductive efforts are similar.

Our study has two limitations: (1) we were unable to compare oxidative stress between breeding and non-breeding females due to limited sample sizes and (2) we did not measure long-term oxidative stress indicators, such as antioxidant enzyme levels. To gain a complete understanding of how oxidative stress influences survival and reproduction in Cape ground squirrels, future research should include longitudinal studies with long-term markers.

Overall, our findings offer a valuable reference for understanding how oxidative stress could decline with age as a result of survival-based selection pressures, thereby enhancing lifetime reproductive success in this species. Our study contributes to a broader understanding of how physiological processes like oxidative stress impact reproductive success and longevity in Cape ground squirrels and potentially other species.

## Figures and Tables

**Table 1:** The generalized linear model was used to analyze oxidative markers—total oxidant status (TOS), total antioxidant activity (TAC), and oxidative stress index (OSI)—for male and female Cape ground squirrels (*Xerus inauris*), with age (years) and body condition (body condition—residuals of size/mass regression) as fixed factors before backward selection based on the Akaike Information Criterion (AIC). Significance at * *p* < 0.05, *** *p* < 0.001, ns as not significant.

Initial Model	Variables Kept After Backward Selection	Estimate	Standard Error	Statistic	*p*-Value
TOS~Sex * body condition * Age, family = Gamma (link = “identity”)	Intercept	6.54726	0.42692	15.336	***
	Sex Male	1.40032	0.32581	4.298	***
	Age	−0.40856	0.07558	−5.405	***
TAC~Sex * Age * body condition, family = Gaussian (link = “inverse”)	Intercept	6.15444315	0.15386392	39.999	***
	Sex Male	−0.09345367	0.18039687	−0.518	ns
	Age	0.01212761	0.03155852	0.384	ns
	body condition	0.00003935	0.00209290	0.019	ns
	Sex Male * Age	0.00864730	0.04158859	0.208	ns
	Sex Male * body condition	0.00097065	0.00246685	0.393	ns
	Age * body condition	0.00019369	0.00037647	0.514	ns
	Sex Male * Age * body condition	0.00041114	0.00055699	−0.738	ns
OSI~Sex * Age * body condition, family = Gamma (link = “identity”)	Intercept	1.07161	0.07580	14.14	***
	Sex Male	0.23510	0.05791	0.05791	***
	Age	−0.06815	0.01336	−5.10	***

**Table 2:** The generalized linear model used to analyze oxidative markers—total oxidant status (TOS), total antioxidant activity (TAC), and oxidative stress index (OSI)—for Male State (natal and band) Cape ground squirrels, with age (years) and body condition (body condition—residuals of size/mass regression) as fixed factors before backward selection based on the Akaike Information Criterion (AIC).

Initial Model	Variables Kept After Backward Selection	Estimate	Standard Error	Statistic (t Value)	*p*-Value
TOS~Male State * Age * body condition, family = Gamma (link = “identity”)	Intercept	8.6543	0.7614	11.366	***
	Male State Natal	−1.0468	−1.0468	−2.151	*
	Age	−0.4404	0.1484	−2.969	**
TAC~Male State * Age * body condition, family = Gaussian (link = “identity”)	Intercept	6.25230	0.16555	37.767	***
	Male State Natal	−0.42375	−0.42375	−1.902	ns
	Age	−0.02071	0.03613	−0.573	ns
	Male State Natal * Age	0.12071	0.06233	1.937	ns
OSI~Male State * Age * body condition, family = Gamma (link = “identity”)	Intercept	1.43497	0.15288	9.386	***
	Male State Natal	−0.16921	0.09748	−1.736	ns
	Age	−0.07723	0.02966	−2.604	*

Significance at * *p* < 0.05, ** *p* < 0.01, *** *p* < 0.001, ns as not significant.

## Data Availability

Data are contained within the article or Supplementary Materials.

## Supplementary Materials

The following supporting information can be downloaded at: https://www.mdpi.com/article/10.3390/antiox13111401/s1. Table S1: The linear regression model used to generate the residuals that estimate body condition.
