# Supplementary material for: Oxidative Stress in an African Ground Squirrel, a Case of Healthy Aging and Reproduction
Source: Antioxidants (Basel). 2024 Nov 15;13(11):1401. doi: 10.3390/antiox13111401 (PMC11591065; doi:10.3390/antiox13111401)
Supplement: Supplementary file 1 [file antioxidants-13-01401-s001.zip › Supplementary Material Final 10102024.pdf]

Table S1

Body condition regression model

Supplementary Table S1. The linear regression model used to generate the residuals that estimate body condition.

**Regression statistics**	
R^2	0.05939
Adjusted R^2	0.04345
DF	1 and 59
F statistic	3.725
p-value	0.05841

	**Estimate**	**Standard error**	**Statistic**	**p (<0.05)**
Intercept	443.347	128.732	3.444	0.00106
Spine length	13.981	7.244	1.930	0.05841
